# Prospective Assessment of Daily Patterns of Mood-Related Symptoms

**DOI:** 10.3389/fpsyt.2018.00370

**Published:** 2018-08-21

**Authors:** Luísa K. Pilz, Alicia Carissimi, Ana Paula Francisco, Melissa A. B. Oliveira, Anastasiya Slyepchenko, Kristina Epifano, Luciene L. S. Garay, Raul C. Fabris, Marina Scop, David L. Streiner, Maria Paz Hidalgo, Benicio N. Frey

**Affiliations:** ^1^Laboratório de Cronobiologia e Sono do Hospital de Clínicas de Porto Alegre (HCPA), Universidade Federal do Rio Grande do Sul, Porto Alegre, Brazil; ^2^Programa de Pós-Graduação em Psiquiatria e Ciências do Comportamento—Faculdade de Medicina, Universidade Federal do Rio Grande do Sul, Porto Alegre, Brazil; ^3^MiNDS Neuroscience Graduate Program, McMaster University, Hamilton, ON, Canada; ^4^Mood Disorders Program and Women's Health Concerns Clinic, St. Joseph's Healthcare, Hamilton, ON, Canada; ^5^Department of Psychology, Neuroscience and Behaviour, McMaster University, Hamilton, ON, Canada; ^6^Department of Psychiatry and Behavioural Neurosciences, McMaster University, Hamilton, ON, Canada

**Keywords:** chronobiology, circadian rhythms, clinical assessment, mood, mental health, self-report questionnaire

## Abstract

**Background:** The Mood Rhythm Instrument (MRI) is a new self-report questionnaire that aims to assess, the presence, and timing of daily patterns of mood-related symptoms. Here, we examined the reliability of the MRI against a prospective daily investigation over the course of 15 days. As a secondary aim, we examined whether the number of items with a perceived daily pattern correlated with severity of depressive symptoms and psychological well-being.

**Methods:** Thirty-two participants recruited from the general population were asked to prospectively fill out a daily version of the MRI (MRI-d) for 15 days. On the 16th day, they filled out the MRI, the Beck Depression Inventory (BDI) and the World Health Organization 5-item well-being index (WHO-5).

**Results:** The MRI showed high agreement with the MRI-d, which suggests that the MRI is a valid tool to assess daily patterns of mood symptoms. The number of mood symptoms perceived as having daily peaks correlated positively with BDI scores and negatively with WHO-5 scores.

**Conclusions:** The MRI might be a valid tool to investigate the presence of daily patterns and the timing of mood-related factors.The MRI does not seem to be influenced by recall or recency biases. Future studies should test the usefulness of this new clinical instrument in individuals with mood disorders, as well as its ability to detect changes in the daily timing of mood symptoms before and after treatment.

## Introduction

Several lines of evidence highlight the mechanistic and phenomenological links between mood symptoms and circadian rhythms (1–3). For instance, the presence of certain polymorphisms in clock genes has been associated with vulnerability and clinical manifestations of mood disorders (4). Animal studies have shown that reduced expression of *Bmal1* in the suprachiasmatic nuclei led to depressive- and anxiety-like behaviors (5). In addition, it has been well established that sleep and circadian disruptions are very prevalent in individuals with major mood disorders such as major depression and bipolar disorder (6–8). Chronotherapies targeting the synchronization of biological rhythms, such as bright light therapy, interpersonal, and social rhythm therapy, sleep deprivation and sleep hygiene, are useful tools in the management of mood disorders (9–12).

Despite the well-established link between alterations in circadian rhythms and the development and clinical presentation of mood disorders, the majority of previous studies have focused on the circadian fluctuation of sleep/appetite patterns, hormonal levels, and sexual/social behaviors. Little is known about circadian fluctuations of psychological symptoms such as sadness, irritability, and mood swings. A better understanding of the circadian rhythmicity of mood symptoms will help to identify individuals whose severity of mood symptoms follow an altered circadian rhythm, which can ultimately guide more accurate treatment decisions. To address this gap, we have recently developed the Mood Rhythm Instrument (MRI). The MRI is a self-report questionnaire that assesses the presence of unimodal daily patterns (i.e., rhythmicity with a peak every 24 h) and the peak timing of physiological and behavioral variables across affective, cognitive, and somatic domains that are often altered in mood disorders in the last 15 days.

The MRI has been validated in Brazilian Portuguese (13) and in Spanish (14), and the English version is currently being validated in Canada. In a large study using the MRI to examine community samples (*N* = 708), we have recently shown that the presence of daily patterns of specific MRI items was significantly associated with higher risk for psychiatric disorders (15). Results also suggested that the timing of some items or the phase angle differences between them might also be related to risk for psychiatry disorders (15). However, one of the main limitations of self-reported questionnaires is the reliance on recall when reporting the presence and severity of past/recent symptoms (e.g., “*past week*”, “*last 15 days*”). Thus, in this study, we aimed to test the reliability of the MRI against a prospective daily investigation over the course of 15 days. We hypothesized that the MRI would have a fair-to-good agreement with a daily version of the MRI (MRI-d). As a secondary aim, we tested whether the number of MRI items where a daily pattern was perceived correlated with severity of depressive symptoms and psychological well-being.

## Materials and methods

### Participants and procedures

Study participants were recruited from the general population through convenience-snowball sampling between November-December 2017 (16). They were asked to fill out a daily version of the MRI (MRI-d) for 15 days. To ensure participants compliance, we met them on day 8 to collect the first week of the MRI-d data and handed the material for the last 7 days. On the 15th day, the remaining MRI-d material was collected, and they were asked to complete the MRI, Beck Depression Inventory [BDI, Beck et al. (17)], the World Health Organization 5-item well-being index [WHO-5, Bech (18); de Souza and Hidalgo (19)] on the next day. Paper-based questionnaires were used. All participants gave their informed consent. The study was approved by the Ethics Committee of Hospital de Clínicas de Porto Alegre (#15-0539 GPPG/HCPA) and was conducted in accordance with the Declaration of Helsinki.

### Instruments

#### Mood rhythm instrument (MRI)

The MRI is a self-report questionnaire that assesses the presence of daily patterns and the peak timing of its 15 items in the previous 15 days. Each MRI item represents a mood-related physiological/behavioral variable. For each item, two questions are asked: (1) whether each item has had a daily peak in the past 15 days (yes/no; *dichotomous variable*) and (2) only for the questions that were answered “yes,” when that peak was (*time variable*).

#### Mood rhythm instrument diary (MRI-d)

The daily version of the MRI-d consists of the same 15 items from the MRI, however participants are asked to answer the questions on a daily basis instead of “in the last 15 days.”

#### Beck depression inventory (BDI)

The BDI is a 21-item self-report questionnaire that assesses severity of current depressive symptoms (17). In this study, the validated Brazilian-Portuguese version was used (20). The BDI is one of the most widely used self-report instruments in the study of severity of depressive symptoms, with high internal consistency in both psychiatric and non-psychiatric samples (21). Higher BDI scores indicate higher severity of depressive symptoms.

#### World health organization 5-item well-being index (WHO-5)

The WHO-5 is a 5-item self-report questionnaire that assesses psychological well-being over the last 2 weeks (19). The WHO-5 has high internal consistency and has been used in several studies as a screening for depression (22). The higher the score, the better the perceived psychological well-being.

### Statistical analysis

Initially, we calculated the mode of each dichotomous MRI-d variable for each participant across the 15 days of the prospective daily charting (*dichotomous variable*). The agreement rates between the MRI and MRI-d items were determined by calculating the proportion of participants for whom the response on the MRI agreed with the mode of the same variables in the MRI-d. Agreement rates < 0.4 were considered poor, 0.4-0.59 were fair, 0.6–0.74 were good, and ≥0.75 were considered excellent (23). In order to assess potential memory bias due to recency effects, the agreement rates were calculated between the MRI and the cumulative mode of the last 3, 5, 7, 11, and 13 days of the MRI-d. If a recency bias was present, the agreement rates would be higher on the days closer to the second study visit (when participants filled out the MRI).

Similarly, we calculated participants' median times for each MRI-d variable using data from the days where timing was reported in the prospective diary (*time variable*). The time differences between the peaks reported in the MRI and MRI-d were calculated across the 15 days, and across the cumulative median of the last 3, 5, 7, 11, and 13 days in order to assess possible recency bias. If a recency bias was present, the difference between the peaks reported on the MRI and the median peaks of the MRI-d would be significantly lower on the days closer to when the MRI was filled. All time variables were tested for normality using the Shapiro-Wilk test. We tested the correlation between the median MRI-d and MRI variables using Pearson's or Spearman's correlation as appropriate.

Finally, we summed the number of dichotomous items scored “yes” for each of the participants and correlated the number of items where a daily pattern was perceived with BDI and WHO-5 scores using Spearman and Pearson correlations, respectively. IBM SPSS Statistics 24 (IBM, NY, US) and GraphPad 6 (GraphPad Software, La Jolla, US) were used for data analysis. GraphPad 6 and El Temps (Antoni Díez-Noguera, Barcelona, ES) were used to plot linear and angular data, respectively.

## Results

### Participants

Thirty-two individuals completed the MRI, MRI-d, WHO-5, and BDI questionnaires. The mean age of the study participants was 35.3 ± 15.9 (age range: 21–71); 63% of the participants were women; and the mean number of years of education was 15.6 ± 2.6. The majority of the participants (88%) were currently working or studying.

### MRI-d: presence of a daily peak - differences between weekdays and weekends

**Figure 1:**
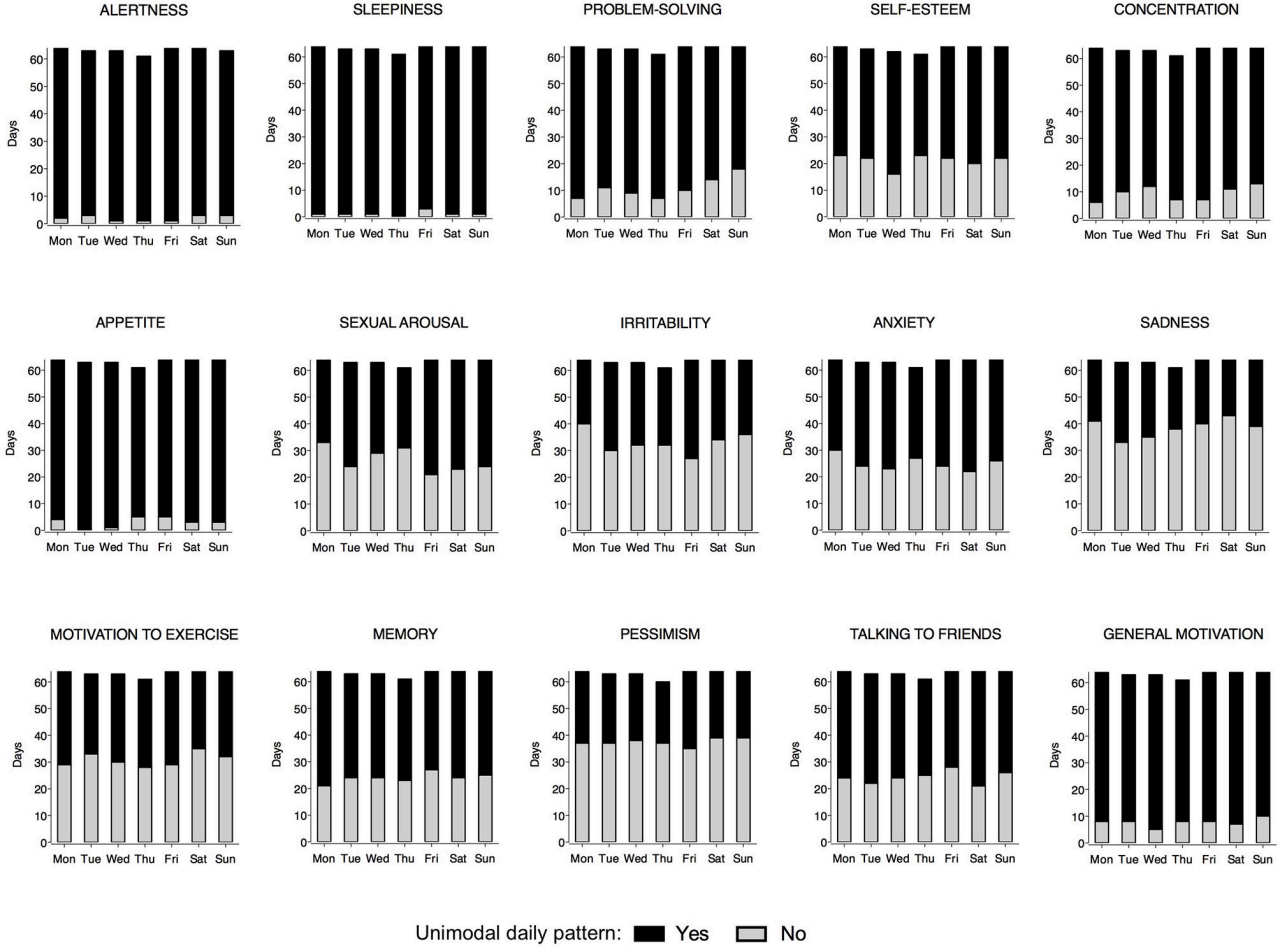
Frequency of days reported as having a peak by weekday for each Mood Rhythm Instrument diary (MRI-d) item. The black portion of the bars represents days where a peak was reported, whereas the gray portion represents days where a peak was not reported. For each MRI-d item, *y-axis*, sum of days from all participants; *x-axis*, weekday. No difference was detected between weekdays vs. weekends (Chi-square).

Figure 1 shows the number of days where participants reported experiencing a peak for each MRI item. Overall, the vast majority of participants reported peaks in cognitive symptoms such as alertness, problem-solving and concentration, as well as in somatic symptoms, such as sleepiness and appetite. A high frequency of perceived daily patterns was also seen in general motivation. On the other hand, peaks in affective symptoms such as sadness, pessimism or irritability were not as often reported. There were no differences between weekdays and weekends in any of the items (*p*> *0.05; Chi-square test*).

**Figure 2:**
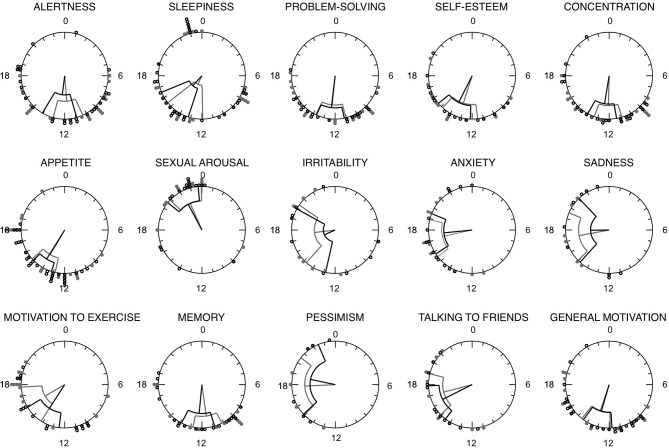
Rayleigh plots for each Mood Rhythm Instrument diary (MRI-d) item time variable. Circles represent the 24 h day. Circles along the outermost circumference represent individuals timing: full gray circles represent the median timing of reported peaks on weekdays, whereas open circles represent the median timing of reported peaks on weekends. Fiducial limits are represented.

Figure 2 shows the distribution of the MRI and MRI-d time variables on weekdays and weekends. Most individuals reported a morning peak for cognitive items (alertness, memory, concentration, problem-solving) and an afternoon/evening peak for affective items (irritability, anxiety, sadness, pessimism). Sexual arousal consistently peaked late at night, whereas sleepiness had a more variable distribution with peaks not only early in the morning and late at night but also after lunchtime. No significant differences were detected between weekdays and weekends (*p* > *0.05; paired t-test/Wilcoxon matched pairs signed-rank test*).

### Agreement rates between the MRI and MRI-d: dichotomous variables

**Figure 3:**
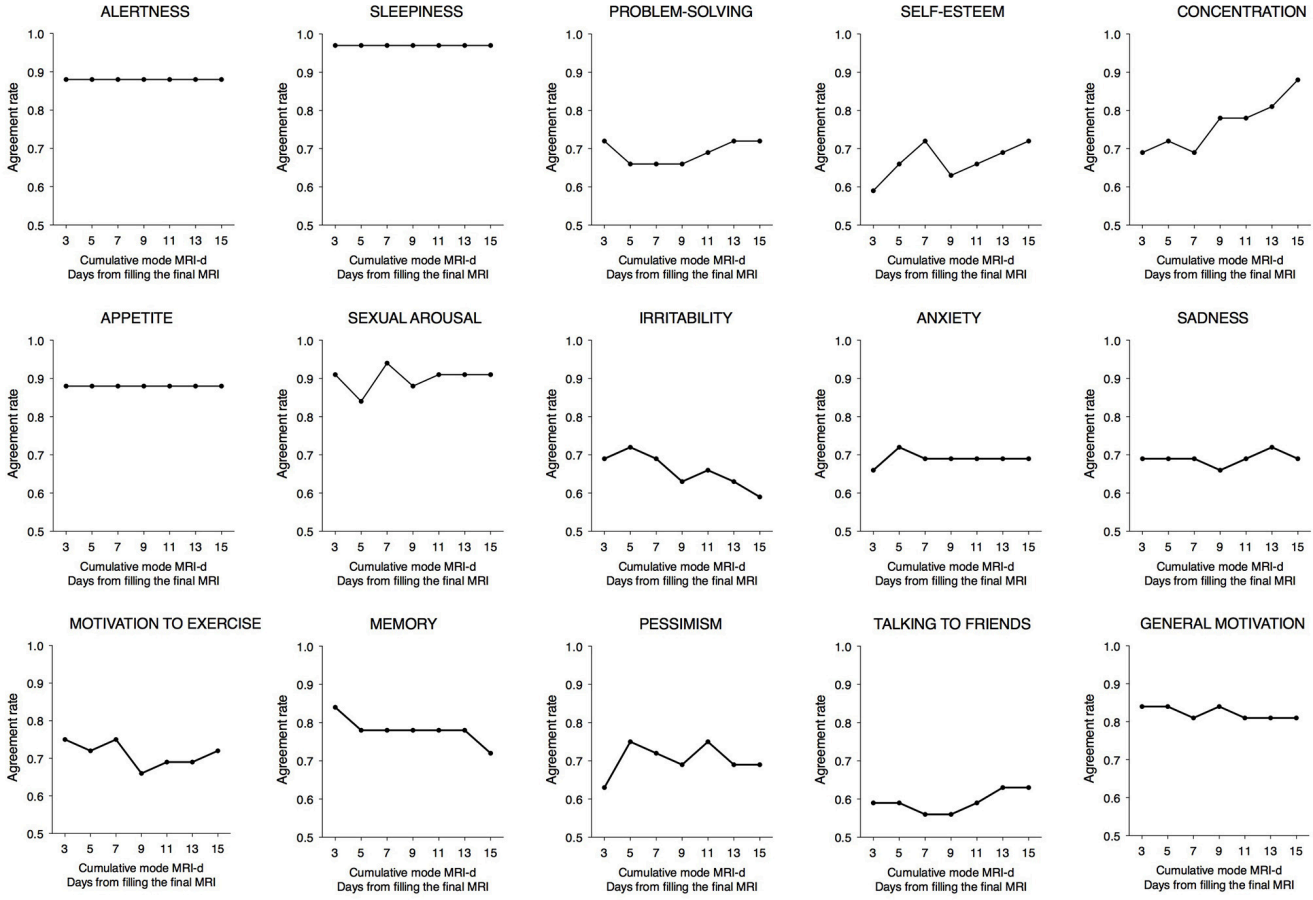
Agreement rates between the Mood Rhythm Instrument (MRI) and the cumulative mode of the last 3, 5, 7, 11, 13, and 15 days from the Mood Rhythm Instrument diary (MRI-d). Dots show group means. Dichotomous data on each item derived from MRI and MRI-d.

Agreement rates for each item are shown in Table 1. All items showed fair to excellent agreement rates, with fair agreement observed for irritability (0.59), good agreement for talking to friends (0.63), and sadness/anxiety (0.69), and excellent agreement observed for sleepiness (0.97), sexual arousal (0.91), and appetite/alertness (0.88). Notably, higher agreement rates were observed in the MRI items where most participants perceived having a daily pattern, whereas lower agreement rates were observed for items that fewer participants reported as having a 24 h peak. Figure 3 shows the agreement rates between the MRI and the cumulative mode of the last 3, 5, 7, 11, 13, and 15 days from the MRI-d. We did not observe an overall effect of recency bias, with the exception of irritability (Figure 3).

**Table 1:** Agreement rates between the MRI and MRI-d.

	**Agreement rate^#^**	**MRI_diff_ (h)^##^**	**MRId median vs. MRI**^**###**^	
	**Peak: y/n**	**Time of peak**	***r***	***n***
Alertness	0.88	−0.65 (−1.63 – 0.33)	0.82^****^	28
Sleepiness	0.97	−0.75 (−1.89 – 0.39)	0.80^****^	31
Problem-solving	0.74	−0.34 (−1.16 – 0.48)	0.82^****^	22
Self-esteem	0.72	−0.02 (−2.59 – 2.56)	0.54^*^	15
Concentration	0.78	−0.34 (−1.28 – 0.60)	0.75^****^	25
Appetite	0.88	−0.48 (−1.18 – 0.22)	0.77^****^	29
Sexual Arousal	0.91	−0.33 (−3.46 – 2.80)	0.56^*^	18
Irritability	0.59	−1.95 (−4.38 – 0.48)	0.80^**^	10
Anxiety	0.69	−1.18 (−3.94 – 1.58)	0.46^+^	14
Sadness	0.69	−0.58 (−3.90 – 2.73)	0.53	9
Motivation to exercise	0.72	0.42 (−0.77 – 1.61)	0.72^***^	19
Memory	0.72	−0.56 (−1.32 – 0.19)	0.80^****^	20
Pessimism	0.69	1.29 (−4.01 – 5.67)	0.39	7
Talking to friends	0.63	0.54 (−0.88 – 1.96)	0.68^*^	12
General Motivation	0.81	−0.64 (−1.86 – 0.58)	0.56^**^	25

# MRI vs. MRI-d;

## MRI_diff_: average MRI-d days median—MRI (95% CI);

### *Correlation coefficient of MRI-d median vs. MRI. Pearson: self-esteem, irritability, anxiety, pessimism, talking to friends. Spearman: alertness, sleepiness, problem solving, concentration, appetite, sexual arousal, sadness, physical exercise, memory, general motivation*.

* *p < 0.05*,

** *p < 0.01*,

*** *p < 0.001*,

**** *p < 0.0001*,

+ *p < 0.10*.

### Agreement between the MRI and MRI-d: time variables

**Figure 4:**
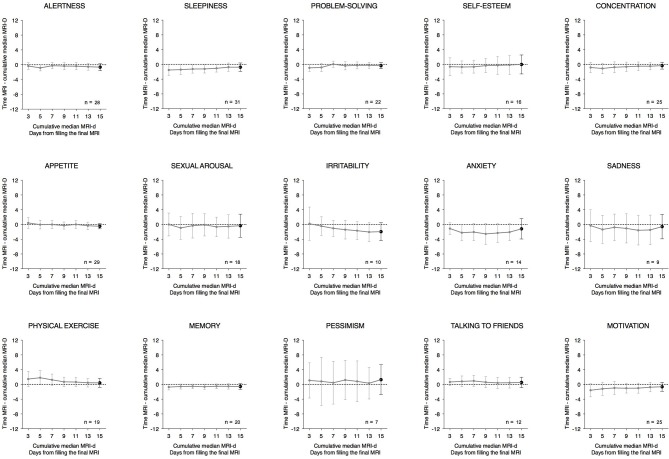
Time differences between the Mood Rhythm Instrument (MRI) and the cumulative median of the last 3, 5, 7, 11, 13, and 15 days from the Mood Rhythm Instrument diary (MRI-d). Dots show group means and whiskers represent 95% confidence intervals. Time data on each item derived from MRI and MRI-d.

The time differences between the peaks reported for each item of the MRI and MRI-d are shown in Table 1 and in Bland-Altman plots (Supplementary Figure 1). For the majority of items, the average difference was less than an hour, suggesting high agreement between the prospectively- and retrospectively-recorded questionnaires. Interestingly, items related to affective symptoms—irritability, anxiety, and pessimism—were the only ones with an average difference greater than an hour. Table 1 also shows the correlation coefficients between the median time variables of the MRI and MRI-d. With the exception of the affective symptoms of pessimism and anxiety, all other items had correlations ≥0.5, which further indicates high concordance between the MRI and MRI-d. Figure 4 depicts the time differences between the MRI and the cumulative median of the last 3, 5, 7, 11, 13, and 15 days of the MRI-d. Similar to the analyses of dichotomous variables, we did not observe an overall effect of recency bias, except for irritability (Figure 4).

### Correlation between the MRI, BDI, and WHO-5

**Figure 5:**
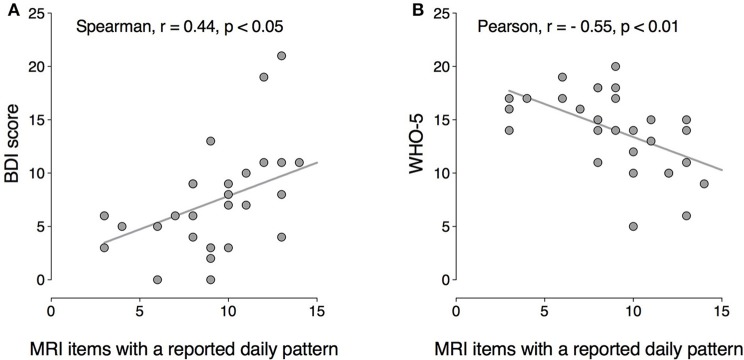
Correlation between the number of mood symptoms with a circadian peak (MRI) and current depressive symptoms (BDI scores), **(A)** and psychological well-being (WHO-5), **(B)** BDI: Spearman *r* = 0.44, *p* < 0.05; WHO-5: Pearson *r* = −0.55, *p* < 0.01.

The number of MRI items with a perceived daily pattern correlated positively with BDI scores (r_S_ = 0.44; *p* < 0.05) and negatively with WHO-5 scores (r_P_ = −0.55; *p* < 0.01; Figure 5). These results suggest that the higher the number of variables perceived as having a daily pattern, the greater the severity of current depressive symptoms and the lower the perceived psychological well-being.

## Discussion

The main finding of this study is that the retrospectively-scored MRI displayed high agreement with the prospectively-recorded MRI-d, which suggests that the MRI is a valid tool to assess rhythmicity—daily patterns and timing—of mood disorders-related factors. In addition, with the exception of “irritability,” none of the other MRI item scores were affected by recency bias. Regarding the dichotomous variables where participants answered whether or not they perceived MRI items to peak every 24 h, we found fair to excellent agreement rates between the MRI-d and the MRI. Irritability was the only item where we observed potential recency bias and, not surprisingly, this item also had the lowest agreement rate between the MRI and MRI-d. As for the time variables, where participants reported the time when the mood-related factors typically peak, the average difference between the MRI and MRI-d was less than an hour for most items except for irritability, anxiety, and pessimism. All correlations between MRI-d and MRI were ≥0.5, except for anxiety and pessimism. Together, these results suggest that the timing of affective/emotional symptoms such as irritability, anxiety and pessimism may be reported with somewhat less accuracy (fair-to-good agreement) as compared with cognitive/somatic mood symptoms (excellent agreement). These results are consistent with prior evidence showing that participants also tend to exaggerate the intensity of positive and negative mood on retrospectively administered scales, as compared to reports obtained daily (24). Overall, retrospective recall of past symptoms of mental illness is subject to recall bias, particularly in the long term, where underestimation of past symptoms and poor recall of previous mood episodes is very common (25). Here it is worth remembering that, contrary to most clinical questionnaires that assess the intensity/severity of symptoms over a period of time, the MRI assesses a different dimension of mood symptoms: if and what time the mood symptoms peak within the day. Our findings suggest that this type of information might be less subject to recency bias.

Interestingly, we found that higher number of mood symptoms perceived as having a daily pattern correlated with higher severity of current depressive symptoms and poorer psychological well-being. These results are consistent with previous evidence showing that depressed outpatients with diurnal mood variation present more severe symptoms and are more likely to meet criteria for melancholic depression (26). On the other hand, hospitalized depressed patients showed no association between severity of mood symptoms and diurnal mood variation (27). Although our study was conducted in the general population, our results are consistent with some of the above evidence in depressed populations suggesting that the perception of rhythmic patterns of mood symptoms is higher in individuals with more severe depressive symptoms. Notably, Wefelmeyer and Kuhs (28) observed that the diurnal variation of mood in healthy individuals was almost exclusively attributed to external circumstances or their own activities, whereas in melancholic patients they occurred spontaneously in more than half cases. Haug and Wirz-Justice (29) proposed that there might be an underlying circadian rhythm of mood masked by daily life events in healthy subjects (28).

The limitations of this study should be considered. We are aware that the MRI does not assess individuals' rhythmicity in a strict sense. Rather, the MRI assesses the subjective perception of recurrent daily peaks of the individual's mood symptoms. Importantly, the MRI is being developed to test its potential utility in clinical settings, such as in the investigation chronodisruption in psychiatric disorders (30), notwithstanding the limitations of self-reported questionnaires alike. Also, our sample size was relatively small, especially for some of the analyses of time variables. However, this was to be expected, because participants should only report a time variable for the items they perceive as having a peak. The fact that the sample size of the analyses of time variables will almost always be smaller should be borne in mind. Notwithstanding this, the sample size was large enough to provide an accurate estimate of the agreement between the MRI and the MRI-d, which was the primary objective of the study. In addition, we were able to show that the MRI is not significantly subject to recency bias. Another limitation was that, due to our sampling method, our study was conducted on a relatively homogeneous sample with a high level of education, which may limit the external validity of our results. Further, we did not recruit a sample of individuals with diagnosed major depressive disorder and, therefore, we do not know if our results are also applicable to individuals with clinical depression. The fact that subjects filled out the MRI shortly after filling out the MRI-d for 15 days could have enhanced the recollection of the items assessed. However, participants were not aware that the aim of the study was to compare the agreement between these questionnaires, and had we asked the participants to fill out the MRI long after the prospective daily charting we would have lost the 15-day window.

In conclusion, we found a high agreement between the retrospectively-scored MRI and the prospectively-recorded MRI-d, indicating that the MRI is a valid tool to investigate the perceived 24 h peak and timing of mood symptoms. We also found that the MRI was not influenced by recency bias. Future studies should test the usefulness of this new clinical instrument in individuals with mood disorders, as well as its ability to detect changes in the daily patterns/timing of mood symptoms before and after treatment.

## Authors contributions

DS, MH, and BF designed the study. LP, AC, AF, MO, RF, and MS collected and organized the data. LP, AC, MO, DS, MH, and BF were involved in data analysis. LP, AC, AF, MO, AS, KE, BF, and MH wrote the first draft of the manuscript. All authors read, revised and approved the final manuscript.

### Conflict of interest statement

The authors declare that the research was conducted in the absence of any commercial or financial relationships that could be construed as a potential conflict of interest.

## Supplementary material

Supplementary Figure 1 Bland-Altman plots showing the difference between the Mood Rhythm Instrument (MRI) and the median time of the Mood Rhythm Instrument diary (MRI-d) on y-axis and the MRI-d median on x-axis. Orange lines represent 1.96 standard deviations of the mean difference. Blue lines represent the average difference (estimated bias).
