# Supplementary material for: Prospective Assessment of Daily Patterns of Mood-Related Symptoms
Source: Front Psychiatry. 2018 Aug 21;9:370. doi: 10.3389/fpsyt.2018.00370 (PMC6110875; doi:10.3389/fpsyt.2018.00370)
Supplement: Supplementary Figure 1 — Bland-Altman plots showing the difference between the Mood Rhythm Instrument (MRI) and the median time of the Mood Rhythm Instrument diary (MRI-d) on y-axis and the MRI-d median on x-axis. Orange lines represent 1.96 standard deviations of the mean difference. Blue lines represent the average difference (estimated bias). [file Data_Sheet_1.PDF]

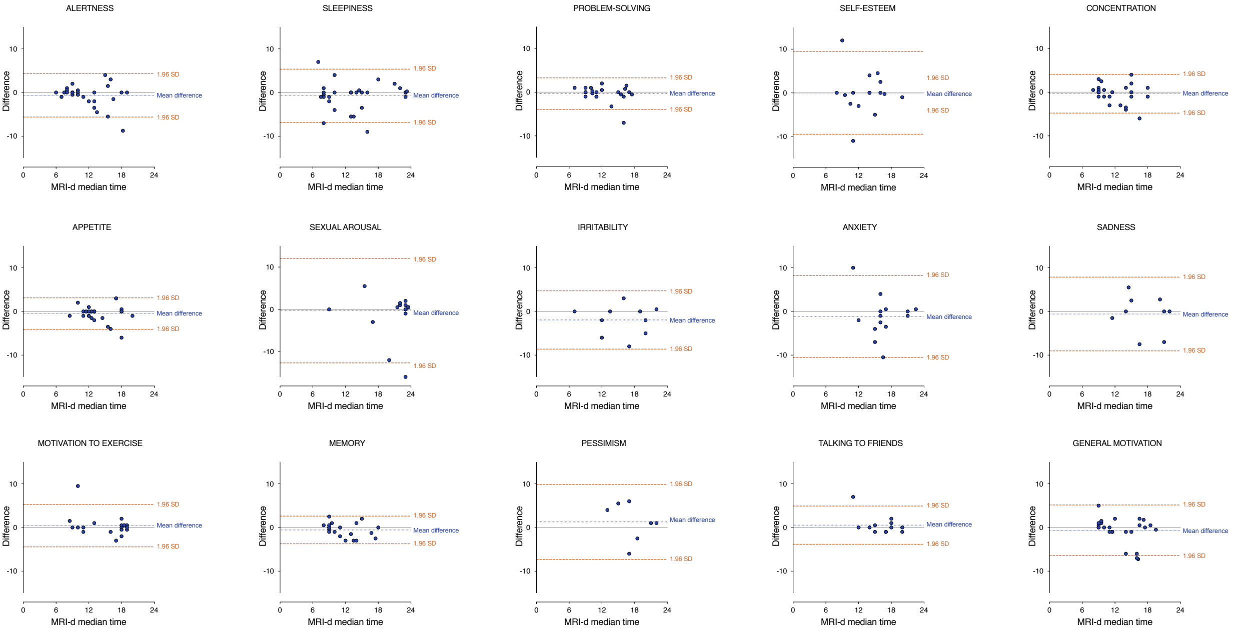

ALERTNESS
SLEEPINESS
PROBLEM-SOLVING
SELF-ESTEEM
CONCENTRATION
APPETITE
SEXUAL AROUSAL
IRRITABILITY
ANXIETY
SADNESS
MOTIVATION TO EXERCISE
MEMORY
PESSIMISM
TALKING TO FRIENDS
GENERAL MOTIVATION
Difference
10
0
-10
1.96 SD
Mean difference
0
6
12
18
24
MRI-d median time
